# Comparison of Behavior-Related Features in the MMSE Sentence in Behavioral Variant Frontotemporal Dementia and Alzheimer’s Disease

**DOI:** 10.3389/fnagi.2021.733153

**Published:** 2021-08-31

**Authors:** Ramiro Ruiz-Garcia, Soojung Yu, Lauryn Richardson, Angela Roberts, Stephen Pasternak, Chloe Stewart, Elizabeth Finger

**Affiliations:** ^1^Clinical Neurological Sciences, Schulich School of Medicine and Dentistry, University of Western Ontario, London, ON, Canada; ^2^Cognitive Neurology and Alzheimer Research Centre, Schulich School of Medicine and Dentistry, University of Western Ontario, London, ON, Canada; ^3^Pepper School of Communication Sciences and Disorders, Northwestern University, Evanston, IL, United States

**Keywords:** bvFTD, Alzheimer’s disease, behavior, Mini-Mental State Examination sentence, writing content

## Abstract

**Background**: Despite the ubiquity of cognitive assessments using the MMSE, there has been little investigation of currently unscored features of the MMSE sentence item relevant to behavior and language in patients with behavioral variant Frontotemporal Dementia (bvFTD) and Alzheimer’s disease (AD).

**Objective**: To describe and compare the unscored content and grammar elements of the MMSE sentence item in patients with bvFTD and AD.

**Methods**: Categorization of predefined content and grammar elements of the MMSE sentence was performed by two blinded raters in patients with bvFTD (*n* = 74) and AD (*n* = 84). Chi-square and ANCOVAs were conducted to identify differences between the diagnostic groups. A multinomial logistic regression analysis was conducted to determine whether these features aid in the prediction of diagnosis of bvFTD or AD.

**Results**: A higher proportion of patients with bvFTD wrote sentences addressed to the examiner (22.7% vs. 4.7%, *X*^2^ = 11.272, *p* = 0.001) and about interpersonal relationships (35.3% vs. 16.0%, *X*^2^ = 10.139, *p* = 0.017) in comparison to those with AD. The number of words written was lower in patients with AD and was positively correlated with lower total MMSE scores in AD but not in bvFTD (AD: *r* = 0.370, *p* < 0.001; FTD: *r* = 0.209, *p* = 0.07). Assessment of the MMSE sentence content and grammar variables did not add to the prediction bvFTD or AD diagnosis beyond the variance explained by age and total MoCA score.

**Conclusions**: Patients with bvFTD and AD showed differences in aspects of the content of the written MMSE sentence item, though these differences did not aid in the diagnosis prediction.

## Introduction

In patients with behavioral variant Frontotemporal Dementia (bvFTD), scores on standard cognitive tests are often preserved, particularly in the early stages of the disease. However, it has been noted that qualitative aspects of cognitive testing are often abnormal in this population (Thompson et al., 2005) and are valuable for differentiation of dementia subtypes (Blair et al., 2006). Patients with bvFTD frequently score normally on the commonly used Mini Mental Status Examination (MMSE; Folstein et al., 1975) at the initial stages of the disease (Tan et al., 2013). However, information that may be gained from qualitative analysis of emotional aspects and linguistic elements of the MMSE written sentence task has been largely unexplored. It has been anecdotally observed that patients with different behavioral disturbances, for example during mania or depression, frequently write sentences with behaviorally relevant content, though empiric investigations of this phenomenon are limited.

bvFTD is a neurodegenerative disease characterized by behavioral changes secondary to apathy and empathy deficits, disinhibition, ritualistic behaviors, impulsivity, and executive cognitive impairment (Rascovsky et al., 2011). Although speech and language impairments have been explored in this population (Hardy et al., 2016; Geraudie et al., 2021), to our knowledge, there are no previous reports examining whether behavioral symptoms related to disinhibition and empathy may be detectable in written language samples from patients with bvFTD. While language, including writing, generally has been considered preserved in bvFTD, a subset of patients may develop language deficits, and many will display impaired word phonemic fluency (Rohrer et al., 2008). Further, as highlighted in a recent review, some patients with bvFTD also show language impairment in lexico-semantic, orthographic, and prosody domains (Geraudie et al., 2021). Further, as the MMSE is frequently used by primary health care providers and in other screening environments, whether the content of the MMSE sentence may help to differentiate patients with bvFTD from those with Alzheimer’s disease (AD) yet has not been examined. In non-language variants of AD, language is generally preserved in the early stages of the disease, though some patients may display word finding difficulties and anomia (Ferris and Farlow, 2013).

The MMSE is one of the most popular cognitive screening tests and has been validated in several countries around the world (Ismail et al., 2010). One item evaluates a sentence written by the patient and awards 1 point if: (1) it is written spontaneously to the instruction “please write a sentence for me”; (2) contains a subject and a verb; and (3) is sensible. Content and other aspects of grammar are not evaluated. Prior studies have explored a limited number of unscored elements of the MMSE sentence in different geriatric populations. Findings have included an association between fewer words and lower total MMSE scores in general geriatric population patients (McCarthy et al., 2004), and in patients with FTD, Vascular dementia, AD, and Parkinson’s disease in comparison with healthy control and MCI population (Corallo et al., 2019). In the same study, an association between the absence of abstract thinking and lower MMSE scores was found in Vascular Dementia and FTD patients, while the Parkinson’s Disease population had increased frequency of concrete sentences; in general, patients with any kind of dementia displayed poorer abstraction in comparison to the healthy control and MCI groups. In another study performed in a general geriatric clinic, no correlations between the number of words and total MMSE scores were found, but higher scores on the 15-item Geriatric Depression Screening Scale (GDS) were correlated with shorter sentences and negative emotional polarity content (Press et al., 2012). Finally, in a study including patients with AD, Vascular dementia, Lewy body dementia, and unspecified dementia, a correlation was found between negative emotional polarity in the sentence and lower quality of life (Sniatecki et al., 2017).

The aim of this study was to characterize and compare the content and qualitative aspects of the MMSE sentence in patients with a clinical diagnosis of possible or probable bvFTD or AD. We hypothesized that in comparison to patients with AD, patients with bvFTD would show qualitative differences in the content of the MMSE sentence related to behavioral symptoms of bvFTD that potentially could be ascertained from the sentence alone (without the benefit of the behavioral observations such as impulsivity or ritualistic behaviors during the writing of the sentence). Thus, we focussed on content reflective of disinhibition and reduced empathy. We examined whether such differences would add to the diagnostic accuracy of a clinical diagnosis of bvFTD vs. AD, beyond that predicted by routine information typically present in primary health care settings (age, gender, MoCA total score), and therefore support the utility of considering one or more of the currently unscored MMSE sentence elements in triage and referral decisions.

## Materials and Methods

### Population

The study sample was extracted retrospectively from the Cognitive Neurology and Alzheimer Research Centre Database in London, Ontario, Canada, from our records from January 2002 to September 2020. Inclusion criteria were a diagnosis of possible or probable bvFTD (Rascovsky et al., 2011) or AD (McKhann et al., 2011) and an available MMSE test including completion of the sentence item. Patients with structural brain lesions (tumor or stroke), patients with a previous diagnosis of schizophrenia or bipolar disorder with psychotic features, and patients who did not speak English as their first language were excluded. For the patients included in this study, the diagnosis of bvFTD or AD was based on a detailed history, neurologic examination, cognitive testing, brain imaging, and in some cases, genetic testing. In the clinic, given the availability of more extensive cognitive testing across the disease-relevant domains, as brief screening tests, the MMSE and MoCA are not the basis for diagnosis between bvFTD and AD but are used as markers of severity, particularly for provincially mandated reported of driving concerns, and for longitudinal assessments. Clinical and cognitive testing data was obtained from the evaluation when a diagnosis of possible or probable AD or bvFTD was initially made. Neurological exam and diagnosis were performed by behavioral neurologists. The study was approved by the University of Western Ontario human subjects research ethics board (#R-11-510) and written informed consent was obtained from all participants. The study adhered to the guidelines of the Declaration of Helsinki.

### Cognitive Screening Batteries and MMSE Sentence Exploration

Cognitive testing was performed by a trained psychometrician. The cognitive evaluation included the complete MMSE, Montreal Cognitive Assessment (MoCA; Nasreddine et al., 2005), and Beck Depression Inventory (BDI) total score (Beck et al., 1961), and typically also included immediate and delayed recall (adapted from the River Mead test), Trails A and B, naming from the Western Aphasia Battery or Boston naming test (15 items), letter and semantic fluency, and clock drawing.

To analyze the MMSE sentence, behaviural and grammatical variables were defined (see Table 1). Behavioral variables included: (a) Emotional polarity, coded as, negative emotion, neutral, or positive emotion; (b) Empathy, coded as empathic, neutral, or non-empathic sentence; (c) Abstraction, defined as the presence of ideas or concepts without physical referents (e.g., sentences about morality, love, etc.); (d) Disinhibition, defined as failure to suppress inappropriate information according to the clinical context; (e) Perseverations, defined as sentences with content related to other MMSE sections (example: “close your eyes”); and (f) Sentence addressed to the examiner. The behavioral variables (c–f) were coded as present or absent. Grammar variables evaluated included the number of words, nouns, verbs, adjectives, pronouns, adverbs, prepositions, grammatical form errors, spelling errors, and lexico-semantic errors. We also included allographic elements of case [normal, only upper case, only lower case, or a mixture of lower and upper case (e.g., ToDay is A good Day)] and font (cursive or printing letter or a mixture). As only a few sentences contained prepositions, these variables were binary coded (0 vs. ≥1 preposition). Grammatical form variables included: the presence of appropriate use of syntactic conjunctions, tenses, conditionals, subordinate clauses, and passive constructions (Boschi et al., 2017). Binary coding was used for grammatical form, spelling errors, lexico-semantic errors, font, and case variables. Finally, the four most common topics observed in the sentences were selected and categorized as: (1) Interpersonal relationships (example: “I love my wife”); (2) Self-descriptive interests (example: “I like to compost”), (3) Life events (example: “I went to see the doctor today”) and (4) Weather (example “It is a sunny day”).

**Table 1:** Definitions and examples of content variables.

Content variable	Definition
Empathy	Identifying with others’ feeling states. Patients with a lack of empathy display a diminished response to others’ feelings and a diminished social interest or personal warmth, e.g., “I want to leave now”.
Abstraction	Abstraction: Presence of ideas or concepts without physical referents. such as sentences about love, morality, democracy, freedom, etc. Lack of abstraction: sentences refer to objects that are available to the senses, e.g., “the grass is green”.
Disinhibition	Sentences were categorized as disinhibited if the content represented a failure to suppress inappropriate information according to the clinical context. e.g., “I need new hair”
Emotional Polarity	Refers to the affective charge included in the sentence. Negative emotion polarity e.g., “Today is a bad day”. Positive emotion polarity e.g., “Today is a wonderful day”. Neutral e.g., “My name is Mike”.
Sentence addressed to examiner	Refers to the direction of content to the evaluator. e.g., “You are a nice girl.”
Perseverations	Sentences with content related to other MMSE sections, e.g., “No, if’s, and’s or but’s”, “Close your eyes”.

All behavioral, topic, and grammar variables were rated by two independent raters blinded to diagnosis and the study hypothesis. Raters only had access to the MMSE sentence. Following the rating of the initial 30 participants, kappa statistics were performed, confirming inter-rater reliability (kappa values >0.60) for the content variables and topics, except for the perseveration content variable which had a kappa value less than 0.60 (see Supplementary Table A). After retraining and consensus scoring on points of discrepancy, raters completed the sentence scoring for all participants. The mean rating for grammar variables was used in the final analysis. For categorical variables, rating discrepancies were reviewed by the raters and a consensus rating was obtained according to previous training and definitions.

### Statistical Analysis

Analyses were performed using SPSS version 26.0 (IBM, Armonk, NY, United States). Differences in demographics and cognitive testing scores between groups were determined by *X*^2^-tests for categorical variables and *t*-tests for continuous variables. For the sentence variables, *X*^2^-tests were conducted for categorical variables, and ANCOVAs including age and MMSE total score as covariates were conducted for continuous variables. Pearson correlations were conducted to assess the relationship between the number of words and the MMSE total score. Two-tailed *p*-values ≤0.05 were considered statistically significant.

Finally, to determine if consideration of the MMSE sentence variables improved the prediction of FTD vs. AD dementia subtype beyond that obtained from age and cognitive screening test scores on the MoCA, the sentence variables found to be statistically significant in the initial analysis were included in a multinomial logistic regression model. Additionally, years of education and MoCA total score were included in the model as covariates. The total score on the MoCA test was selected as it better reflects disease severity across bvFTD and AD groups, particularly at early stages of bvFTD where it is more likely to detect impairments than the MMSE (Coleman et al., 2016).

## Results

### Demographic and Cognitive Testing Data

One hundred and fifty-eight patients met the inclusion criteria. Participants in the bvFTD (*n* = 74) and AD groups (*n* = 84) were similar in gender distribution, years of education, years of clinical symptoms before diagnosis, and BDI total scores (see Table 2, part A). As expected, the mean age at the time of diagnosis was higher for the patients with AD compared to bvFTD. Scores on the cognitive testing screens were lower in the AD group than in bvFTD. Ten participants in the bvFTD group (13.5% of the sample) had a definite diagnosis due to a known mutation (six C9orf72, two MAPT, and two GRN). From the AD group, one patient had a PSEN 1 mutation.

**Table 2:** Demographic and clinical characteristics of bvFTD and AD groups.

	bvFTD (*n* = 74)	AD (*n* = 84)	*t* value	*CI 95% (lower-upper)*	*p*-value
**(A) Demographic and clinical characteristics**
Female, *n*(%)	31 (40.8%)	48 (55.8%)	3.645*	-	0.061
Age at time of diagnosis, mean (SD)	65 (9.78)	70.77 (9.74)	3.710	2.700–8.848	**<0.001**
Years of education, mean (SD)	13.05 (3.17)	12.88 (4.08)	−0.296	−1.319–0.976	0.764
Years of clinical symptoms, mean (SD)	3.56 (2.48)	2.87 (2.14)	−1.469	−1.624–0.242	0.145
MMSE score, mean (SD)	24.43 (6.09)	22.45 (5.58)	−2.159	−3.793 to −0.179	**0.033**
MoCA score, mean (SD)	19.94 (5.97)	14.39 (5.64)	−5.469	−7.550 to −3.539	**0.001**
Beck depression inventory, mean (SD)	11.87 (21.6)	11.22 (22.7)	−0.130	−10.666–9.355	0.897
**(B) Grammar elements**			***F* value**	
N. Words, mean (SD)	6.16 (3.30)	5.43 (2.16)	0.257	-	0.613**
N. Nouns, mean (SD)	1.64 (1.10)	1.40 (0.80)	0.898	-	0.345**
N. Verbs, mean (SD)	1.42 (1.00)	1.26 (0.86)	0.186	-	0.667**
N. Adjectives, mean (SD)	0.55 (0.63)	0.51 (0.58)	0.101	-	0.751**
N. Pronouns, mean (SD)	0.82 (0.75)	0.70 (0.61)	0.248	-	0.619**
N. Adverbs, mean (SD)	0.36 (0.56)	0.30 (0.47)	0.008	-	0.927**
N. of subjects with ≥ 1 Preposition (%)	27, (36%)	19, (22.6%)	3.450^++^	-	0.063^+^
Grammatical form errors, *n*(%)	19, (25%)	16, (18.8%)	0.900^++^	-	0.343^+^
Spelling errors, *n*(%)	20, (26.3%)	20, (23.3%)	0.203^++^	-	0.652^+^
Lexical-semantic errors, *n*(%)	9, (12%)	7, (8.2%)	2.259^++^	-	0.520^+^
Case, mixed features, *n*(%)	43, (56.6%)	55, (64%)	1.813^++^	-	0.612
Font, cursive letter, *n* (%)	46, (53.5%)	33, (43.4%)	1.648^++^	-	0.439

AD, Alzheimer Disease; bvFTD, behavioral variant Frontotemporal Dementia; MMSE, Mini Mental State Examination; MoCA, Montreal Cognitive Assessment Battery. Part A. Demographic and clinical characteristics. Student *t*-test was conducted for variables with exception of gender; for the later *X^2^ test was performed. Adjusted *p*-values ≤0.05 were considered statistically significant and are shown in bold. *Part B. Grammar elements* of the sentence in bvFTD and AD groups. N, number. **P-value of ANCOVA analysis using covariates (MMSE total score and age at first evaluation); ^+^*p* value for chi square analysis. ^++^*X*^2^value.

### Sentence Elements

**Figure 1:**
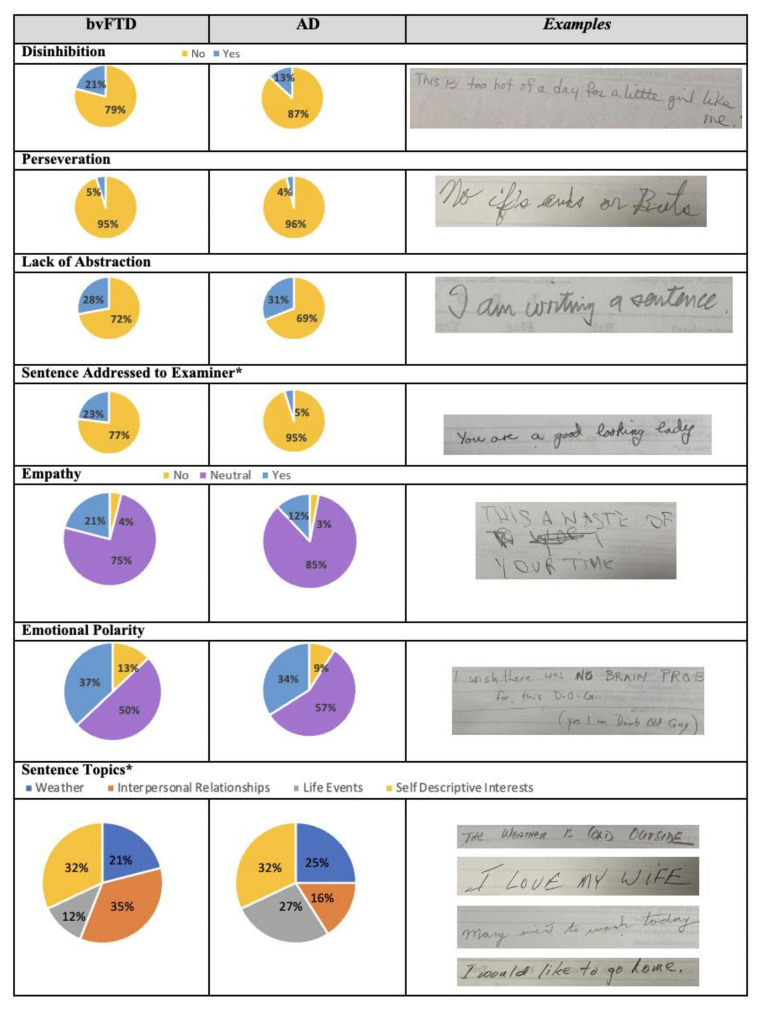
Frequency and examples of sentence content and topics of the MMSE sentence in bvFTD and AD. bvFTD, behavioral variant Frontotemporal Dementia; AD, Alzheimer disease. *p-value <0.05. Examples in the last column are for: presence of disinhibition, presence of perseverations, lack of abstraction, presence of sentence addressed to the examiner, lack of empathy, and negative emotion polarity. Examples for sentence topics are displayed in the following order: weather, interpersonal relationships, life events, and self-descriptive interests.

The frequency of content variables and between group comparisons are shown in Figure 1. A higher proportion of patients with bvFTD wrote sentences addressed to the examiner in comparison to those with AD (22.7% vs. 4.7%, *X*^2^ = 11.272, *p* = 0.001). There were no significant differences in the frequency of other content elements including disinhibition (21.3% vs. 12.9%, *X*^2^ = 2.000 *p* = 0.157), perseverations (5.3% vs. 5.9%, *X*^2^ = 0.230, *p* = 0.880), non-empathic sentences (4.0% vs. 3.5%, *X*^2^ = 2.770 *p* = 0.250), negative emotional polarity (13.3% vs. 9.3%, *X*^2^ = 1.168, *p* = 0.558), and lack of abstraction (28.0% vs. 31.0%, *X*^2^ = 0.166, *p* = 0.684).

The proportion of sentence topics differed between the groups (*X*^2^ = 10.139, *p* = 0.017; Figure 1). A greater number of patients with AD wrote sentences related to life events (27.2% vs. 11.8%,) and weather (24.7% vs. 20.6%) in comparison to the bvFTD group. A greater proportion of patients with bvFTD wrote about interpersonal relationships (35.3% vs. 16.0%), while a similar proportion was observed for self-descriptive interests (32.2% vs. 32.1%).

**Table 3:** Logistic regression model of MMSE sentence elements to predict bvFTD vs. AD diagnosis.

bvFTD*	b (SE)	*p*-value	Odds Ratio	95% CI Lower	95% CI Upper
Years of education	−0.154 (0.071)	**0.031**	0.857	0.745	0.986
MoCA score	0.189 (0.045)	**<0.001**	1.208	1.106	1.320
Interpersonal relationships	0.713 (0.713)	0.318	2.040	0.504	8.260
Timely life events	−0.283 (0.651)	0.664	0.753	0.210	2.700
Self-descriptive interests	0.797 (0.590)	0.176	2.219	0.669	7.049
Weather	0^b^				
Absence of sentence addressed to examiner	−1.532 (0.909)	0.092	0.216	0.036	1.282

Note. *R*^2^ = 0.27 (Cox and Snell), 0.37 (Nagelkerke). Model $X_{\left(6\right)}^{2}$ = 37.944, *p* < 0.001. *Reference category is Alzheimer Disease. p-values <0.05 were considered statistically significant and are shown in bold. b: this parameter was set to zero because it was redundant in the response category “Topics”. b, unstandardized beta coefficient.

The total number of words written, classification of words written, grammatical form variables, lexico-semantic errors, and allographic elements (font and case) did not differ significantly between the groups (see Table 2, part B). The total number of words written was positively correlated with the MMSE total score for patients with AD (*r* = 0.370, CI 95% 0.197–0.506, *p* < 0.001), though the correlation did not reach significance in the bvFTD group (*r* = 0.209, CI 95% 0.072–0.344, *p* = 0.07).

The MMSE sentence variables showing significant group differences as described above (sentence addressed to the examiner and sentence topics) were then entered into a multinomial logistic regression, with years of education and MoCA total score as covariates (see Table 3). The model was significant and predicted 37% of the diagnosis variance between the diagnostic groups (Nagelkerke *R*^2^ = 0.37). However, none of the content variables contributed significantly to the group membership prediction.

## Discussion

To the best of our knowledge, this is the first study to examine whether abnormal behavioral symptoms of bvFTD are reflected in the content of the MMSE sentence item. Contrary to our initial hypothesis, when comparing sentence content from patients with bvFTD to those with AD, both groups overlapped considerably in the majority of MMSE sentence variables of interest. While a greater proportion of patients with bvFTD wrote sentences addressed to the examiner and wrote about interpersonal relationships, differences in the frequencies of these variables did not contribute significantly to predicting bvFTD vs. AD diagnosis.

The patterns observed are in keeping with the classic symptom profiles of bvFTD and AD. The larger proportion of patients with bvFTD addressing their sentence to the evaluator may reflect an environmental dependence-like phenomenon of behavioral disinhibition, where the evaluator is the most novel and salient stimulus in the room (Ghosh et al., 2013). Patients with AD wrote mainly about life events, likely reflecting the heightened representation of relatively preserved long–term memory processes in the context of short–term memory deficits (Weintraub et al., 2012), as most of the sentences related to this topic were descriptions of remote events or routines involving implicit memory processes.

Although these trends fit with predictions, the considerable overlap in the sentence content across the bvFTD and AD groups may reflect the mild stage at which the task was completed. The sentences included in this study were obtained from patients at their first presentation to the cognitive neurology clinic, typically during the initial stages of the disease, when disinhibition and related symptoms are mild or moderate. Further, in the early stages of bvFTD, the highly structured environment in a hospital clinic and cognitive testing room are known to influence the expression of behavioral changes, as patients are often able to conform to behavioral norms for limited periods of time (Snowden et al., 2001). Alternatively, it is possible that written language expression might not be a useful or reliable way to detect behavioral disinhibition, as the act of writing usually is not followed by an instant reinforcement and therefore represents an effortful “pure cognitive” task. Future prospective exploration of qualitative aspects of writing in bvFTD patients could include other features that may reflect impulsivity, such as the time spent in completing the task (e.g., less time in impulsive patients), and samples from more naturalistic settings, such as evaluation of email or texting content.

Patients with bvFTD wrote a greater number of words and grammar elements in comparison with the AD group. Patients with AD also had lower total scores in the MMSE, which was correlated with the number of words written. These results are consistent with previous reports in patients with cognitive decline, showing a positive correlation between the number of words in the MMSE sentence and the MMSE total score (McCarthy et al., 2004; Corallo et al., 2019).

Limitations of this study include the retrospective nature and cross-sectional design. While most of the patients were at the initial stages of disease when differences in performance may be more subtle, we considered this stage most relevant to assessing the value of the MMSE sentence task. Patients in the early stages of the disease are the population most commonly evaluated in primary care settings with cognitive screening tools like the MMSE, where quick assessments of aide diagnosis and direction of referrals are most valuable. While we used the MoCA to control potential differences in disease severity, other clinical measures of function and disease severity, beyond the MMSE and MoCA, were not available for much of this retrospective cohort. Additionally, we did not have data from a healthy control group to compare with the patient groups. Finally, although our inter-rater reliability was high for most of the variables, we observed some discrepancies in our evaluations for the content variables including disinhibition, empathy, and perseverations. Further standardization of these subjective elements may be beneficial given the subjective component of qualifying behavioral elements in a sentence.

In conclusion, patients with bvFTD and AD showed differences in aspects of the content of the written MMSE sentence item, though these differences did not aid in the prediction of diagnosis of bvFTD and AD beyond contributions of age and total MoCA scores. Further studies, including a healthy control group and other dementia subtypes, may be helpful to determine whether consideration of content elements of the MMSE sentence may aid in the differentiation of other dementia subtypes.

## Data Availability Statement

The raw data supporting the conclusions of this article will be made available by the authors, without undue reservation.

## Ethics Statement

The studies involving human participants were reviewed and approved by University of Western Ontario human subjects research ethics board (#R-11-510). The patients/participants provided their written informed consent to participate in this study. Written informed consent was obtained from the individual(s) for the publication of any potentially identifiable images or data included in this article.

## Author Contributions

EF, RR-G, SP, AR, and CS designed the study. EF and RR-G analyzed and interpreted the data. SY and LR rated all the variables. EF and SP evaluated all the patients. EF supervised the study. All the authors critically revised the manuscript. All authors contributed to the article and approved the submitted version.

## Conflict of Interest

The authors declare that the research was conducted in the absence of any commercial or financial relationships that could be construed as a potential conflict of interest.

## Publisher’s Note

All claims expressed in this article are solely those of the authors and do not necessarily represent those of their affiliated organizations, or those of the publisher, the editors and the reviewers. Any product that may be evaluated in this article, or claim that may be made by its manufacturer, is not guaranteed or endorsed by the publisher.
