# Supplementary material for: Comparison of Behavior-Related Features in the MMSE Sentence in Behavioral Variant Frontotemporal Dementia and Alzheimer’s Disease
Source: Front Aging Neurosci. 2021 Aug 31;13:733153. doi: 10.3389/fnagi.2021.733153 (PMC8439252; doi:10.3389/fnagi.2021.733153)
Supplement: Supplementary file 1 [file Table_1.DOCX]

Supplementary Material

Comparison of behavior-related features in the MMSE sentence in behavioral variant Frontotemporal dementia and Alzheimer’s disease

Ramiro Ruiz-Garcia ^1,2^, Soojung Yu ^2^, Lauryn Richardson ^2^, Angela Roberts ^3^, Stephen Pasternak ^1,2^, Chloe Stewart ^2^, Elizabeth Finger ^1,2^

^1^Clinical Neurological Sciences, Schulich School of Medicine & Dentistry, University of Western Ontario, London, Ontario, Canada

^2^Cognitive Neurology and Alzheimer Research Centre, Schulich School of Medicine & Dentistry, University of Western Ontario, London, Ontario, Canada

^3^Pepper School of Communication Sciences and Disorders, Northwestern University, Evanston, IL, USA

*** Correspondence to:**Dr. Elizabeth Finger
Elizabeth.Finger@lhsc.on.ca

**Supplementary Table A.** Kappa value of pilot sentences and final scores

| Variable | Kappa value  pilot sentences | Kappa value final ratings | P value |
| --- | --- | --- | --- |
| Empathy | 0.780 | 0.497 | <0.001 |
| Abstraction | 0.792 | 0.824 | <0.001 |
| Disinhibition | 0.672 | 0.310 | <0.001 |
| Emotional Polarity | 0.790 | 0.703 | <0.001 |
| Sentence addressed to examiner | 0.607 | 0.681 | <0.001 |
| Perseverations | 0.403 | 0.413 | <0.001 |
| Topics | 0.619 | 0.627 | <0.001 |

- Note all cases were ratings differed for these categorical variables, raters discussed each discrepancy and agreed on a consensus rating.
